# Simulation analysis of *EGFR* mutation detection: Oncomine Dx target test and AmoyDx panel impact on lung cancer treatment decisions

**DOI:** 10.1038/s41598-024-52006-6

**Published:** 2024-01-18

**Authors:** Yosuke Hirotsu, Takahiro Nakagomi, Yuki Nagakubo, Taichiro Goto, Masao Omata

**Affiliations:** 1 Genome Analysis Center, Yamanashi Central Hospital, 1-1-1 Fujimi, Kofu, Yamanashi 400-8506 Japan; 2 Lung Cancer and Respiratory Disease Center, Yamanashi Central Hospital, 1-1-1 Fujimi, Kofu, Yamanashi Japan; 3 Division of Genetics and Clinical Laboratory, Yamanashi Central Hospital, 1-1-1 Fujimi, Kofu, Yamanashi Japan; 4 Department of Gastroenterology, Yamanashi Central Hospital, 1-1-1 Fujimi, Kofu, Yamanashi Japan; 5 The University of Tokyo, 7-3-1 Hongo, Bunkyo-ku, Tokyo, Japan

**Keywords:** Molecular medicine, Lung cancer

## Abstract

Lung cancer is a leading cause of cancer-related deaths worldwide. Epidermal growth factor receptor (*EGFR*) driver mutations are crucial for treatment decisions for patients with non-small cell lung cancer (NSCLC). This study aimed to assess the differences in *EGFR* mutation detection between two companion diagnostic (CDx) tests—the Oncomine Dx Target Test (ODxTT) and the AmoyDx Pan Lung Cancer PCR Panel—and their impact on treatment applicability. To this end, we used an in-house targeted sequencing dataset of 282 samples from 127 *EGFR*-mutated NSCLC patients to simulate the concordance between the *EGFR* variants targeted by the ODxTT and AmoyDx panel, the oncogenicity of the variants, and their therapeutic potential. Of the 216 *EGFR* mutations identified by the in-house panel, 51% were detectable by both CDx tests, 3% were specific to ODxTT, and 46% were not targeted by either test. Most non-targeted mutations did not have oncogenicity and were located outside exons 18–21. Notably, 95% of the mutations detectable by both tests had potential oncogenicity. Furthermore, among the 96 patients harboring actionable *EGFR* mutations, 97% had mutations detectable by both CDx tests and 1% by ODxTT, while 2% had mutations not covered by either test. These findings suggest that while both CDx tests are effective in detecting almost all actionable *EGFR* mutations, ODxTT provides slightly broader coverage. These results emphasize the importance of selecting appropriate CDx tests to inform treatment decisions for *EGFR*-positive NSCLC patients.

## Introduction

Lung cancer is a highly prevalent and refractory disease worldwide, accounting for a high number of cancer-related deaths^1^. The discovery of driver mutations in the epidermal growth factor receptor (*EGFR*) gene has revolutionized the treatment landscape for a subset of patients with non-small cell lung cancer (NSCLC)^2,3^. *EGFR* mutations occur in approximately 15–20% of NSCLC patients and are detected mainly in exon 19 deletions and the p.L858R mutation in exon 21. These mutations confer sensitivity to EGFR tyrosine kinase inhibitors (TKIs), leading to improved clinical outcomes^4–6^.

EGFR TKIs, including gefitinib, erlotinib, afatinib, and osimertinib, have demonstrated remarkable efficacy in patients harboring sensitizing *EGFR* mutations^7–10^. EGFR TKIs bind to the adenosine triphosphate-binding site within the intracellular domain and inhibit tyrosine kinase activity, leading to perturbation of downstream signaling pathways involved in tumor cell proliferation and survival^11^. EGFR TKIs have been shown to significantly prolong progression-free survival and improve prognosis compared with conventional chemotherapies.

To facilitate treatment decision-making and prolong patient survival, companion diagnostic (CDx) tests are commonly applied in clinical practice for lung cancer patients^12^. The identification of actionable mutations in tumors helps clinicians to select appropriate targeted therapies. In Japan, two widely used CDx tests—the Oncomine Dx Target Test (ODxTT) and the AmoyDx Pan Lung Cancer PCR Panel (hereafter AmoyDx panel)—are available for patients with NSCLC^13–15^. ODxTT is based on next-generation sequencing analysis^16^, while the AmoyDx panel uses multiplex real-time PCR-based assay^15^. Notably, each CDx test analyzes different sites of *EGFR* mutations. To date, ODxTT can detect 146 types of *EGFR* mutations, whereas AmoyDx panel can detect 63 types of *EGFR* mutations at the coding level according to the attached documents^15,17^. Therefore, *EGFR* mutations may be detectable in one test but not in the other. However, no reports have investigated the extent to which these two CDx tests cover *EGFR* mutations in lung cancer specimens.

In this study, we aimed to simulate the differences in *EGFR* mutation detection at the level of nucleotide changes between the ODxTT and AmoyDx panel and assess the impact on treatment applicability. To this end, we used a dataset obtained from the analysis of an in-house lung cancer panel. These simulation data provide useful insights into whether CDx tests detect actionable *EGFR* mutations and report therapeutic potential.

## Results

### *EGFR* mutations in the study cohort

**Figure 1:**
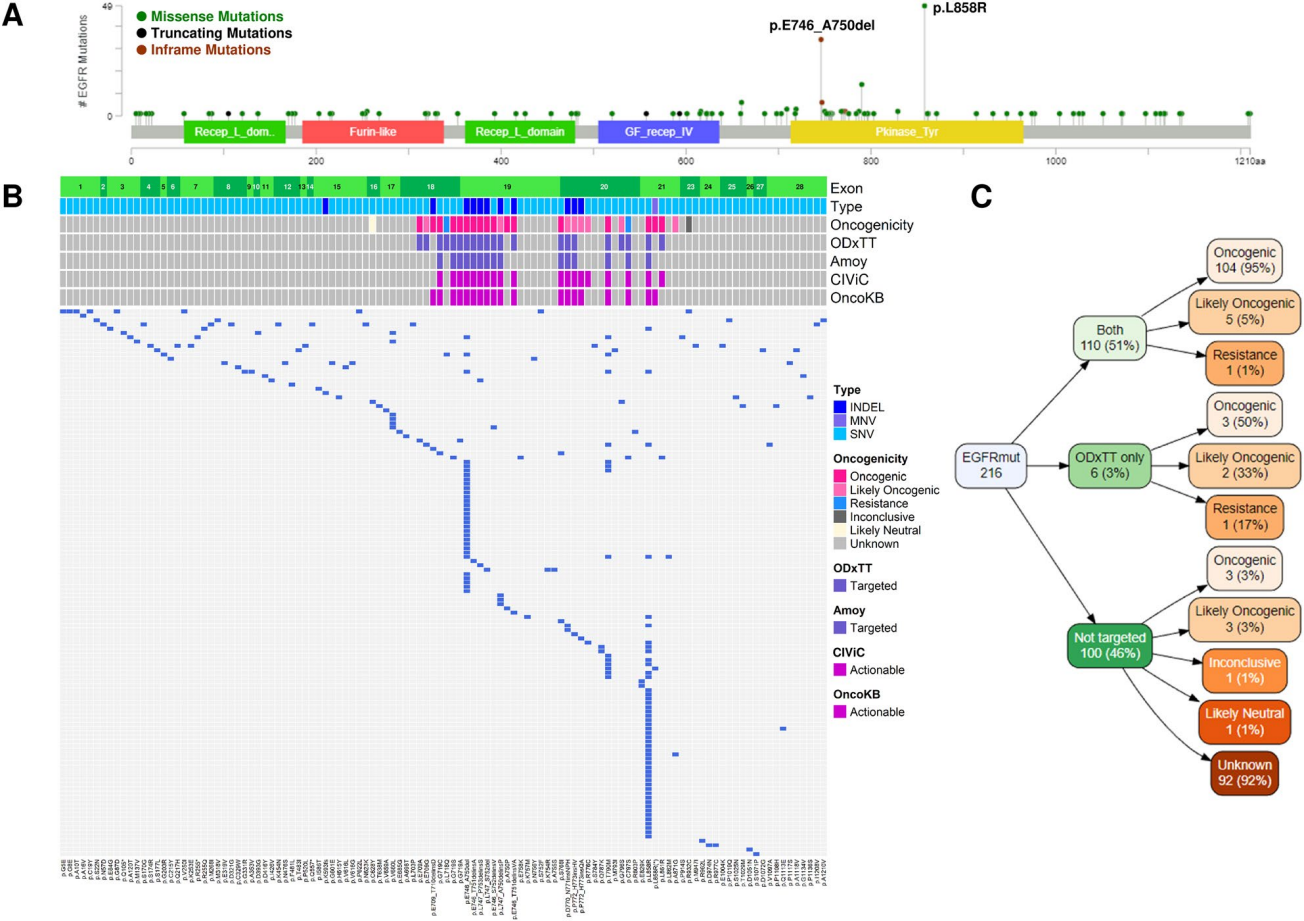
*EGFR* mutations targeted by CDx tests. (**A**) Lollipop plot showing the location of amino acid changes in *EGFR* mutations identified in the in-house panel. Green circles represent missense mutations, black circles represent truncation mutations, and brown circles represent in-frame mutations. The height corresponds to the number of samples in which *EGFR* mutations were detected. (**B**) The heatmap shows a list of *EGFR* mutations in each sample. The vertical axis represents the samples, and the horizontal axis represents the amino acid changes. The top annotations indicate the exon, mutation type, oncogenicity, targeted mutations for ODxTT and AmoyDx panel, mutations registered as actionable in CIViC, and mutations registered as actionable in OncoKB. (**C**) The variable tree diagram categorizes the *EGFR* mutations (total of 216 mutations) into targeted mutations for testing (green layer) and oncogenicity (orange layer).

We performed targeted sequencing using an in-house lung cancer panel covering the entire exon regions of 53 genes and studied 127 NSCLC patients. In these specimens, we identified 216 *EGFR* mutations: 167 missense mutations, 45 in-frame insertion/deletion mutations and 4 truncating mutations (Fig. 1A, Supplementary Table 1). The identified mutations were distributed as follows: 4.2% (9/216) in exon 18, 22% (47/216) in exon 19, 13% (28/216) in exon 20, 25% (54/216) in exon 21, and 36% (78/216) in other exons (Supplementary Table 2). The most frequently detected mutations were p.L858R at 23% (49/216) and p.E746_A750del at 14% (31/216) (Fig. 1A).

### Comparison of ODxTT and AmoyDx panel

Using the dataset of *EGFR* mutations, we performed a simulation to assess the detection rate of the ODxTT and AmoyDx panel. Among the 216 *EGFR* mutations, 110 (51%) were targeted in both CDx tests, 6 (3%) were specific to ODxTT, and 100 (46%) were not included in the target regions of either CDx test (Fig. 1B and Table 1). There were no mutations that were only detected by the AmoyDx panel. All the mutations detectable by the ODxTT and/or AmoyDx panel were located in exons 18–21 (Fig. 1B). However, the mutations not targeted by either CDx test were predominantly found in exons other than exons 18–21 (78%, 78/100) (Fig. 1B, Supplementary Table 3).

**Table 1:** Oncogenicity and actionable potential of *EGFR* mutations targeted by CDx tests.

Characteristic	Both (N = 110)	ODxTT only (N = 6)	Not targeted (N = 100)
Oncogenicity, n (%)			
Oncogenic	104 (95%)	3 (50%)	3 (3%)
Likely oncogenic	5 (5%)	2 (33%)	3 (3%)
Resistance	1 (1%)	1 (17%)	0 (0%)
Inconclusive	0 (0%)	0 (0%)	1 (1%)
Likely neutral	0 (0%)	0 (0%)	1 (1%)
Unknown	0 (0%)	0 (0%)	92 (92%)
CIViC or OncoKB, n (%)			
Therapeutic	110 (100%)	2 (33%)	4 (4%)
Unknown	0	4 (67%)	96 (96%)

Both, targeted by both ODxTT and AmoyDx panel; ODxTT, Oncomine Dx Target Test; CIViC, Clinical Interpretation of Variants in Cancer; OncoKB, a precision oncology knowledge base.

To examine the oncogenicity of *EGFR* mutations, we referred to a precision oncology knowledge base (OncoKB)^18^. Among the 110 mutations detectable by both CDx tests, 104 (95%) were classified as oncogenic, 5 (5%) as likely oncogenic, and 1 (1%) as a resistant mutation (Fig. 1C and Table 1). Among the six mutations detectable only by ODxTT, three (50%) were oncogenic, two (33%) were likely oncogenic, and one (17%) was a resistant mutation (Fig. 1C). The six mutations covered by ODxTT but not by the AmoyDx panel were p.E709A (c.2126A > C), p.E709G (c.2126A > G), p.L718Q (c.2153 T > A), p.E746_T751delinsVA (c.2237_2253delAATTAAGAGAAGCAACAinsTTGCT), p.G796S (c.2386G > A), and p.L861R (c.2582 T > G) (Table 2).

**Table 2:** Summary of *EGFR* mutations that are detectable only by ODxTT or not targeted by both tests.

ID	Exon	Mutation	Coding	Oncogenicity	Category	CIViC or OncoKB
P036	18	p.E709A	c.2126A > C	Oncogenic	ODxTT only	NA
P063	18	p.E709G	c.2126A > G	Likely oncogenic	ODxTT only	NA
P123	18	p.L718Q	c.2153 T > A	Resistance	ODxTT only	NA
P040	19	p.E746_T751delinsVA	c.2237_2253delAATTAAGAGAAGCAACAinsTTGCT	Oncogenic	ODxTT only	Actionable
P022	20	p.G796S	c.2386G > A	Likely oncogenic	ODxTT only	NA
P055 *	21	p.L861R	c.2582 T > G	Oncogenic	ODxTT only	Actionable
P084	19	p.A750P	c.2248G > C	Oncogenic	Not targeted	NA
P117	18	p.E709_T710delinsD	c.2127_2129delAAC	Oncogenic	Not targeted	Actionable
P015	20	p.P772_H773insQA	c.2318_2319insCAGGCG	Likely oncogenic	Not targeted	Actionable
P065 *	20	p.R776C	c.2326C > T	Likely oncogenic	Not targeted	Actionable
P077 *	21	p.L858R(*)	c.2573_2574delTGinsGT	Oncogenic	Not targeted	Actionable
P067	21	p.A871G	c.2612C > G	Likely oncogenic	Not targeted	NA

Asterisk indicates cases with other actionable *EGFR* mutations. ODxTT, Oncomine Dx Target Test; CIViC, Clinical Interpretation of Variants in Cancer; OncoKB, a precision oncology knowledge base.

### Non-target *EGFR* mutations by both ODxTT and AmoyDx panel

The majority of the 100 mutations not included in the target regions of both CDx tests (92/100, 92%) had unknown functional significance (Fig. 1C). However, a small number of mutations were classified as oncogenic (3/100, 3%) or likely oncogenic (3/100, 3%) (Fig. 1C and Table 1). These mutations were p.A750P (c.2248G > C), p.E709_T710delinsD (c.2127_2129delAAC), p.P772_H773insQA (c.2318_2319insCAGGCG), p.R776C (c.2326C > T), p.L858R (c.2573_2574delTGinsGT), and p.A871G (c.2612C > G) (Table 2).

### Actionable *EGFR* mutations covered by ODxTT and AmoyDx panel

**Figure 2:**
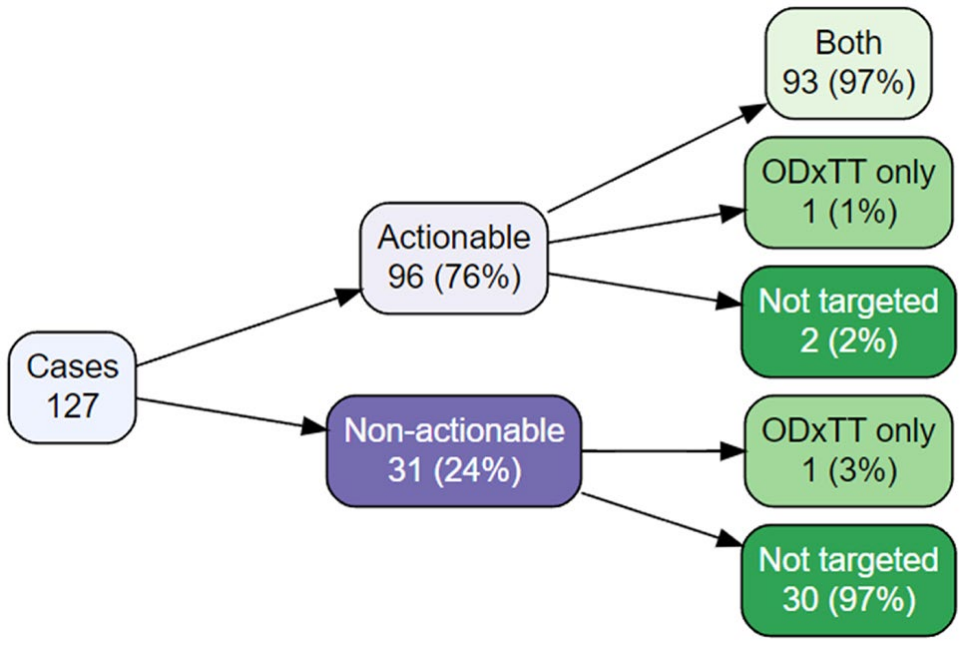
Relationship between actionable mutations covered by CDx tests and clinical cases. Variable tree diagram classifying lung cancer patients (total of 127 cases) with *EGFR* mutations into cases with actionable mutations according to CIViC and OncoKB (purple layer) and cases with targeted mutations for testing (green layer).

To determine whether the *EGFR* mutations included in the target regions of the ODxTT and AmoyDx panel corresponded to therapeutic mutations, we referred to the Clinical Interpretation of Variants in Cancer (CIViC) and OncoKB databases^18,19^. Among a total of 127 cases, 96 (76%) had actionable *EGFR* mutations, while 31 (24%) had non-actionable mutations (Fig. 2).

Among the 96 cases with actionable mutations, 93 (97%) had mutations detectable by both the ODxTT and AmoyDx panel, 1 (1%) had a mutation detectable only by ODxTT (p.E746_T751delinsVA [c.2237_2253delAATTAAGAGAAGCAACAinsTTGCT] in case ID: P040), and 2 (2%) had mutations not included in the target regions of either test (p.E709_T710delinsD [c.2127_2129delAAC] in case ID: P117 and p.P772_H773insQA [c.2318_2319insCAGGCG] in case ID: P015) (Fig. 2 and Table 2). *EGFR* p.E709_T710delinsD (c.2127_2129delAAC) in exon 18 is registered in the Catalogue of Somatic Mutations In Cancer (COSMIC; ID: COSV51779132) and the corresponding drug is afatinib with level 3A bases on OncoKB. *EGFR* p.E709_T710delinsD is classified into P-loop and αC-helix compressing (PACC) subgroups and is sensitive to second-generation TKIs^20^. The exon 20 insertion mutation p.P772_H773insQA (c.2318_2319insCAGGCG) is considered a rare mutation with no COSMIC registration^21^, but the corresponding drugs are amivantamab and mobocertinib with level 1 bases on OncoKB. These results suggested that both CDx tests can lead to EGFR TKI treatment for most patients. However, simulation results showed that actionable mutations could not targeted by the two CDx tests at a frequency of 2% (Fig. 2).

### Impact of *EGFR* co-mutation on treatment applicability

In two patients, we observed the presence of *EGFR* co-mutations, where one mutation was not detected by either CDx test, and the other mutation was detected by both tests and was classified as an actionable mutation (Supplementary Table 4). In case P065, p.L858R (c.2573 T > G)/p.R776C (c.2326C > T) double mutation was identified, while in case P077, p.L858R (c.2573_2574delTGinsGT)/p.T790M (c.2369C > T) double mutation was observed. In these cases, the impact on treatment applicability was not affected because neither one of the detectable mutations represented an actionable alteration. Notably, however, the multi-nucleotide variant (MNV) of p.L858R (c.2573_2574delTGinsGT) that was observed in case P077 was not included in the target regions of both CDx tests. As a result, the therapeutic applicability of these diagnostic tests may be limited when rare *EGFR* mutations are present in samples.

## Discussion

This study showed whether the ODxTT and AmoyDx panel could detect *EGFR* mutations from a real-world specimen from patients with lung cancer. Our analysis simulated that among the 216 *EGFR* mutations, 51% were targeted by both CDx tests, 3% were specific to ODxTT, and 46% were not included in the target regions of either test. All *EGFR* mutations detected by either of the two tests or by ODxTT were located in exons 18–21 and were functionally relevant mutations. In contrast, the majority of mutations not covered by both CDx tests were located outside exons 18–21 and were functionally unknown mutations. There were actionable *EGFR* mutations in 96 patients, of which 93 patients (97%) were targeted by both CDx tests. Therefore, the treatment option of EGFR TKIs can be selected for most NSCLC patients by performing ODxTT and AmoyDx panel. In contrast, addressing the forthcoming challenge involves ensuring the identification of the remaining three cases without overlooking them in CDx tests.

There are rare *EGFR* actionable mutations in NSCLC patients. Notably, this study included the rare *EGFR* p.L858R mutation caused by an MNV (c.2573_2574delTGinsGT) and this MNV is not targeted by either CDx test. Regarding the MNV, we have previously reported that the Kirsten rat sarcoma viral oncogene homolog (*KRAS*) p.Q61K (c.180_181delTCinsAA) was detected in colorectal cancer through next-generation sequencing analysis, but not by a real-time PCR based assay^22^. The AmoyDx panel is based on the amplification refractory mutation system^23^, which uses 3'-prime mismatches of the nucleotide changes. Therefore, the AmoyDx panel possibly can detect both the single nucleotide variant (c.2573 T > G) and the MNV (c.2573_2574delTGinsGT); however, validation is necessary using samples with the MNV because detailed primer information is not disclosed. Therefore, covering rare *EGFR* actionable mutations by CDx tests remains a future challenge.

EGFR TKIs are drugs that can be highly effective for patients with *EGFR*-positive NSCLC. Thus, it is crucial to minimize any potentially missed mutations by the CDx test. In Japan, the ODxTT and AmoyDx panel are the main CDx tests that can analyze multiple genes simultaneously. Either one of these tests is supported by the national health insurance system, but undergoing both tests is not supported. Furthermore, the targeted mutations are different between the two CDx tests, causing concern and frustration when selecting a CDx test. One contributing factor is that the targeted *EGFR* mutations are different, making it unclear how many *EGFR* mutations can be reported to patients. In this study, actionable *EGFR* mutations that could be detected by both CDx tests covered 97% of cases, allowing treatment options to be determined for the majority of patients. However, in 2% of cases, actionable mutations were not detected by either test. The challenge is how to identify these missed cases in first-line treatment. Such discrepancies have also been reported in previous studies^24,25^. To date, ODxTT has expanded the number of detectable variants included in the report through updates to the analysis program. To reduce the number of missed cases, there is a need to expand the scope of reported rare *EGFR* mutations in current next generation sequencing-based analyses or to approve newly developed CDx tests^26,27^.

This study had several limitations. First, this study simulated detectable mutations by the ODxTT and AmoyDx panel using accumulated datasets from our in-house lung cancer panel. Therefore, whether *EGFR* mutations can actually be detected by these two platforms remains uncertain. Second, an increase in *EGFR* mutation datasets is needed to obtain more robust results. In particular, rare mutations in *EGFR* have been reported^28^; therefore, it is necessary to pay attention to whether they are detected by these CDx platforms. Finally, this cohort includes patients who were treated with EGFR-TKIs and have acquired *EGFR* resistance mutations. In fact, the ODxTT and AmoyDx panel are used before first-line treatment for NSCLC. These differences may have influenced the *EGFR* detection rate in this study.

Overall, our findings provide useful information about *EGFR* detection by the ODxTT and AmoyDx panel. Both CDx tests showed efficacy in detecting clinically relevant mutations and there were small differences in their performance in identifying rare *EGFR* mutations. This information is valuable for clinicians in selecting the most appropriate diagnostic test based on the specific needs of each patient with NSCLC.

## Methods

### Patients

In this study, we selected patients with *EGFR*-mutated NSCLC who had undergone targeted sequencing with an in-house lung cancer panel (see below). We used datasets from 282 samples (103 tumor tissues, 34 cytological specimens, 18 plasma, and 127 buffy coats) from 127 patients with NSCLC. This included 63 men (50%) and 64 women (50%) with a median age of 69 years old (interquartile range, 63–75). Written informed consent was obtained from all patients. This study was approved by the Institutional Review Board of the Clinical Research and Genome Research Committee at Yamanashi Central Hospital (G-2018-1) and complied with Declaration of Helsinki principles.

### Sample processing and DNA extraction

Peripheral blood samples were collected and centrifuged to separate buffy coats and plasma. Buffy coats were stored at − 80 °C until DNA extraction. Buffy coat DNA was extracted with the QIAamp DNA Blood Mini QIAcube Kit (Qiagen, Hilden, Germany). The DNA concentration of buffy coats was determined using a NanoDrop 2000 (Thermo Fisher Scientific, Waltham, MA, USA). Plasma DNA was extracted with the MagMax Cell-Free DNA extraction kit on the KingFisher Duo Prime (Thermo Fisher Scientific). Concentration of plasma DNA was determined using the Qubit dsDNA HS Assay Kit and Qubit 3.0 fluorometer (Thermo Fisher Scientific) according to the manufacturer’s instructions.

Tumor tissues were fixed using 10% buffered formalin^29^. Serial 10 μm sections were prepared from formalin-fixed paraffin-embedded (FFPE) tissues, and sections were stained with hematoxylin–eosin and reviewed by a pathologist to check the tumor area. Laser capture microdissection was performed using an Arcturus XT laser microdissection system (Thermo Fisher Scientific). FFPE DNA was extracted using the QIAamp DNA FFPE Tissue Kit (Qiagen), the GeneRead DNA FFPE Kit (Qiagen), and the MagMAX™ FFPE DNA/RNA Ultra Kit (Thermo Fisher Scientific) according to the manufacturer’s instructions. FFPE DNA concentrations were determined using the Qubit® dsDNA HS Assay Kit on a Qubit Fluorometer 3.0 (Thermo Fisher Scientific).

### Targeted sequencing

We used an in-house lung cancer panel targeting 53 genes using IonAmpliseq Designer, as described previously^30^. Briefly, multiplex PCR was performed using the Ion AmpliSeq Library Kit v2.0 or Ion AmpliSeq Library Kit Plus (Thermo Fisher Scientific). Primers were digested with FuPa reagent and then barcoded using Ion Xpress Barcode Adapters. Purification was performed by Agencourt AMPure XP reagents (Beckman Coulter, Brea, CA, USA) using the KingFisher Duo Prime System (Thermo Fisher Scientific). The library concentration was determined using an Ion Library Quantitation Kit. Emulsion PCR and chip loading were performed on the Ion Chef with the Ion PI Hi-Q Chef Kit. Sequencing was performed using the Ion PI Hi-Q Sequencing Kit on the Ion Proton Sequencer (Thermo Fisher Scientific).

Targeted sequencing was also conducted on the Ion Torrent Genexus System in accordance with the manufacturer’s instructions (Thermo Fisher Scientific). DNA concentrations were diluted to 1.1 ng/µL in nuclease-free water. Amplification of DNA was performed using the aforementioned in-house lung cancer panel. The Ion Torrent Genexus Library Strips and Templating Strips were incubated at room temperature for 30 min before being loaded into the sequencer.

### Data analysis

Raw signal data from the sequencing analysis were processed using the standard pipeline in the Torrent Suite Software running on the Torrent Server or in the Genexus Software in the Ion Torrent Genexus System. The data processing pipeline involved signaling processing, base calling, quality score assignment, read alignment, quality control of mapping, and coverage analysis. Following data analysis, the annotation of somatic variants was performed by the Ion Reporter Server System (Thermo Fisher Scientific). To perform tumor–normal pair analysis, we used buffy coat DNA as a normal control for subtraction of germline mutations and to detect somatic variants in tumors. We used the following filtering parameters for variant calling: (i) minimum number of variant allele reads ≥ 10; (ii) coverage depth ≥ 50; (iii) variant allele fraction (VAF) ≥ 0.05; (iv) UCSC Common SNPs = Not In; and (v) Confident Somatic Variants = In.

If the same *EGFR* mutation was identified in multiple samples from a single patient, duplicate mutations were excluded and not used in the subsequent analysis. The evaluation of *EGFR* mutations targeted by the ODxTT (Thermo Fisher Scientific) and AmoyDx Pan Lung Cancer PCR Panel (Amoy Diagnostics Co., Ltd., Xiamen, China) was performed according to the attached documents, which were from Thermo Fisher Scientific and Riken Genesis (Tokyo, Japan) as of June 2023 (Supplementary Table 5). Comparisons with corresponding mutations were conducted based on coding information. Mutations that could be detected by both CDx tests were classified as “Both.” Mutations that could only be detected by ODxTT were categorized as “ODxTT only.” Mutations that were not covered by either test were classified as “not targeted.” We also searched the registration of *EGFR* mutations in COSMIC, version 98 released 23 May 23^31^.

### Oncogenicity and actionable mutations

To evaluate the oncogenicity of mutations, we referred to OncoKB as of June 20, 2023^18^. We searched for the gene name "*EGFR*" and extracted the mutations under the “Annotated Alterations” tab, specifically focusing on the “Oncogenic” category. If the identified mutation corresponded to *EGFR* exon 19 in-frame insertions/deletions and exon 20 in-frame insertions, these mutations were classified as “Likely oncogenic”.

To assess the actionable mutations, we searched both the CIViC and OncoKB databases as of June 20, 2023. We downloaded the tab-separated values (.tsv) file of Clinical Evidence Summaries from the CIViC Data Releases (date: March 1, 2023) and selected entries that included “molecular_profile” with “EGFR,” “disease” with “Lung,” and “therapies” with mentions of corresponding drugs (Supplementary Table 6). Additionally, we downloaded the ‘Actionable Genes’ dataset from OncoKB, selecting “EGFR” as the gene and “Non-Small Cell Lung Cancer” as the cancer type (Supplementary Table 7).

Mutations were categorized as follows: if a corresponding drug was mentioned in either CIViC or OncoKB, it was classified as “actionable”; if no mention was found in either database, it was classified as “non-actionable”. Furthermore, in cases where multiple *EGFR* co-mutations were present in a single patient, if at least one mutation was present in the target lists of both CDx tests, it was categorized as “Both”. If at least one mutation was present in the target list of ODxTT only and did not meet the previous condition, it was classified as “ODxTT only”. If neither condition was met, the case was categorized as “Not targeted”.

### Data processing and visualization

Data processing and visualization in R (version 4.1.1) (http://www.r-project.org/) were also performed using ggplot2 (v3.3.5), ggpubr (v0.4.0), dplyr (v1.0.7), tidyr (v1.1.3), scales (v1.2.1), patchwork (v1.1.1), gtsummary (v1.5.2), flextable (v.0 0.7.0), vtree (v.5.6.5), and ComplexHeatmap (v.2.14.0) packages.

### Supplementary Information

Supplementary Table 1.

Supplementary Table 2.

Supplementary Table 3.

Supplementary Table 4.

Supplementary Table 5.

Supplementary Table 6.

Supplementary Table 7.

## Data Availability

The source data underlying figures and tables are available upon reasonable request from the corresponding author.
